# Supplementary material for: Association between oral microbiome and seven types of cancers in East Asian population: a two-sample Mendelian randomization analysis
Source: Front Mol Biosci. 2023 Nov 21;10:1327893. doi: 10.3389/fmolb.2023.1327893 (PMC10702768; doi:10.3389/fmolb.2023.1327893)
Supplement: Supplementary file 1 [file DataSheet1.ZIP › Supplementary Materials/MR plots of saliva/saliva═╝/ovarian cancer/pheno.3719_to_ovarian cancer_forest.pdf]

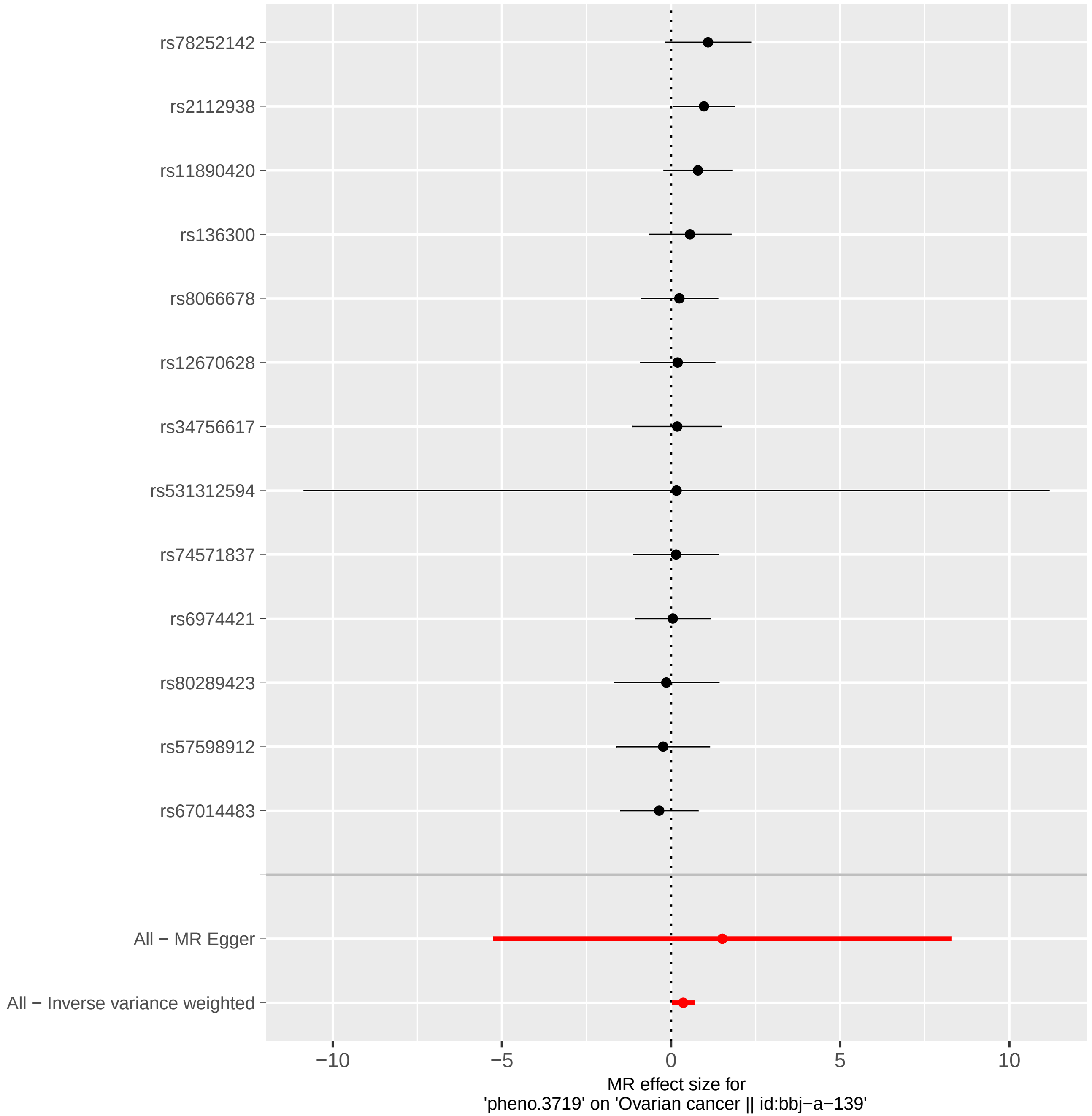
rs78252142
rs2112938
rs11890420
rs136300
rs8066678
rs12670628
rs34756617
rs531312594
rs74571837
rs6974421
rs80289423
rs57598912
rs67014483
All − MR Egger
All − Inverse variance weighted
−10
−5
0
5
10
MR effect size for
'pheno.3719' on 'Ovarian cancer || id:bbj−a−139'
